# Supplementary material for: Inverse-designed diamond photonics
Source: Nat Commun. 2019 Jul 25;10:3309. doi: 10.1038/s41467-019-11343-1 (PMC6658519; doi:10.1038/s41467-019-11343-1)
Supplement: Supplementary file 1 — Supplementary Information [file 41467_2019_11343_MOESM1_ESM.pdf]

Supplementary Material: Inverse-Designed Diamond Photonics

Constantin Dory, Dries Vercruysse,[*] Ki Youl Yang,[*] Neil V. Sapra, Alison E. Rugar, Shuo Sun, Daniil M. Lukin, Alexander Y. Piggott, Jingyuan L. Zhang, Marina Radulaski,[†] Konstantinos G. Lagoudakis,[‡] Logan Su and Jelena Vučković

E. L. Ginzton Laboratory, Stanford University, Stanford, California 94305, USA

[*]These authors contributed equally.
[†]Also at: Electrical and Computer Engineering, University of California, Davis, CA 95616, USA
[‡]Now at: Department of Physics, University of Strathclyde, Glasgow, G4 0NG, UK

Supplementary Note 1: Fabrication methods

In our experiments we use electronic grade diamond from Element Six and grow 200 nm Si_xN_y using low pressure chemical vapor deposition. We then spin coat hydrogen silsesquioxane FOx-16 and define the patterns using electron-beam lithography. With a SF_6, CH_4 and N_2 reactive ion etch (RIE), we transfer the pattern into the Si_xN_y hard mask (Supplementary Figure 1a). With this hard mask, we perform an anisotropic O_2 RIE (Supplementary Figure 1b). Afterwards, we deposit 30 nm of Al_2O_3 via atomic layer deposition (Supplementary Figure 1c), which we then remove from the horizontal planes with a Cl_2, BCl_2 and N_2 RIE (Supplementary Figure 1d). After a short second anisotropic diamond etch to expose the sidewalls of the structure (Supplementary Figure 1e), we quasi-isotropically undercut the structures (Supplementary Figure 1f). During this quasi-isotropic RIE we turn off the forward bias and increase the inductively coupled plasma bias to 3000 W. Due to a preferential etch along $\{110\}$ (Supplementary Figure 1g) we undercut the diamond membrane along these planes. Monitoring the progress of the etch using high voltage scanning electron microscopy, we determine the thickness of the devices. Once the desired thickness is reached we terminate the etch. To remove Si_xN_y and Al_2O_3, we soak the samples in hydrofluoric acid and reveal a suspended structure with rectangular cross section (Supplementary Figure 1h). Figure S2a is a scanning electron micrograph of the fabrication step in Supplementary Figure 1g. The white, rectangular material in the SEM is diamond, covered by Si_xN_y (dark material) and the sides by Al_2O_3 (transparent material). High voltage imaging allows us to measure the thickness of the diamond beneath the Al_2O_3 mask. The bottom surface of the diamond structures is flat and

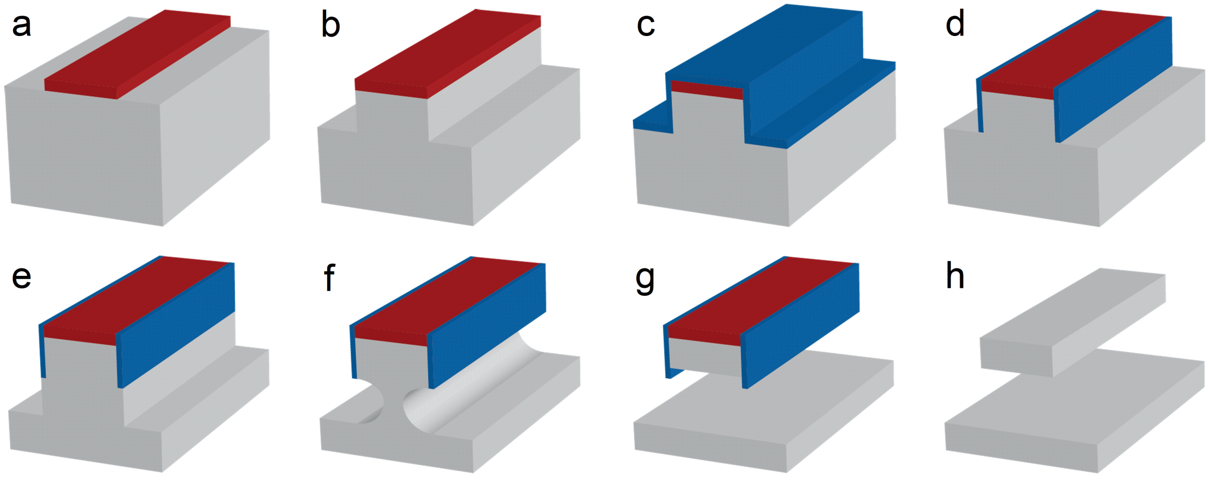

Supplementary Figure 1: **Fabrication protocol for rectangular cross section with quasi-isotropic etching technique.** **(a)** Si_xN_y etch mask (red) on diamond substrate (gray). **(b)** Anisotropic oxygen dry etch of diamond. **(c)** Atomic layer deposition of Al_2O_3 (blue). **(d)** Anisotropic dry etch of Al_2O_3. **(e)** Anisotropic etch of diamond. **(f)** Quasi-isotropic etch of diamond undercuts the structure along the $\{110\}$ facet. **(g)** Etch forms flat bottom surface since the etch rate along $\{001\}$ is slower than along $\{110\}$, which allows for rectangular cross sections. **(h)** Removal of Si_xN_y and Al_2O_3 mask results in suspended nanobeam.

smooth as evidenced from Figure S2b, a SEM of a microdisk that broke off. In Supplementary Figure 2c we present a nanobeam photonic crystal cavity with vertical couplers from top-down view. The fabricated nanobeams in this publication have a width of $w = 400$ nm and a thickness of $h \approx 230$ nm. The lattice constant of the photonic crystal cavities is $a = 234$ nm, and the radii of the holes are $r_x = a \cdot 0.26$ and $r_y = w \cdot 0.21$. The outer ten holes of the photonic crystal are kept constant, while the inner eight holes are tapered down to $0.81 \cdot (r_x, r_y)$ to form a cavity.

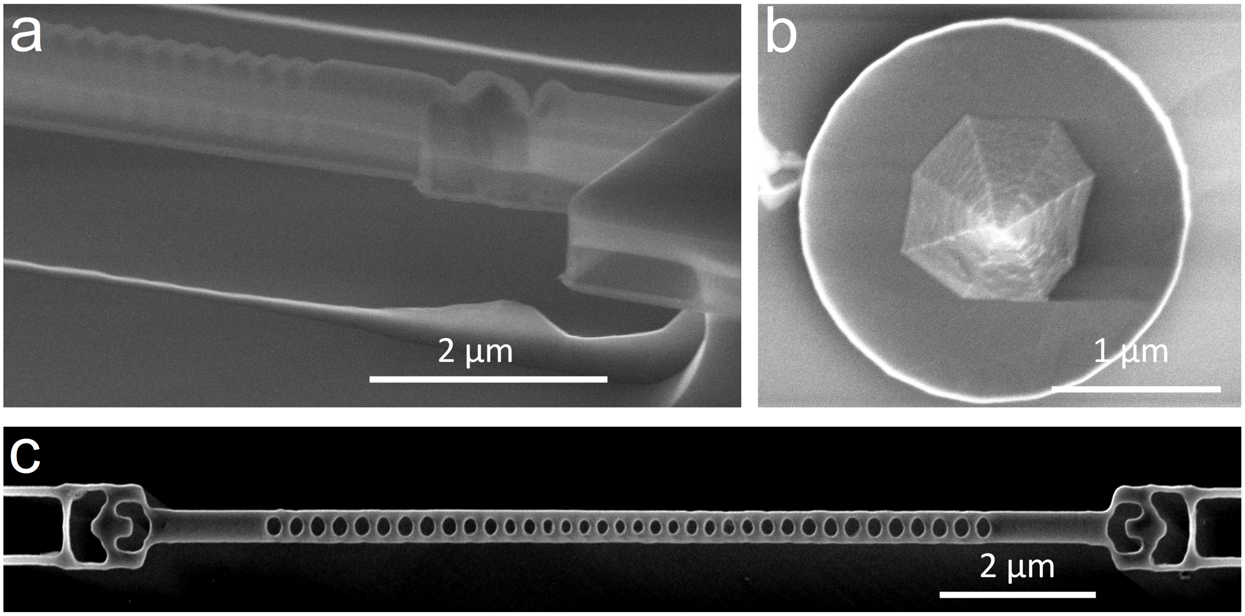

Supplementary Figure 2: **SEM of diamond devices.** **(a)** SEM of a device corresponding to the step in Supplementary Figure S1g. The device (bright material) is protected from the top by Si_xN_y (dark material) and the sides by Al_2O_3 (transparent material). **(b)** Fabricated diamond microdisk that broke off. SEM visualizes the smooth and flat bottom surface. Nanobeam photonic crystal cavity with vertical couplers in **(c)** top-down view.

Supplementary Note 2: Characterization of vertical couplers

We characterize the vertical couplers using the setup depicted in Figure S3. With a half-wave plate and a polarizer we optimize the polarization to be perpendicular to the propagation direction. We measure the power at the beamsplitter, at the cryostat, and at the end of the detection fiber. We characterize the losses of the setup, the wavelength-dependent transmission and reflection ratio of the beamsplitter. We also take the transmission of the objective into account. We assume no losses in the diamond waveguide and no coupling losses at the fiber couplers to calculate a conservative measurement result. Because we use vertical couplers for in- and out-coupling of the light, we cannot state separate efficiencies for in- and out-coupling. We first characterize the coupling efficiency of the vertical couplers into single-mode polarization maintaining fibers (PMF) by using PMF at both the input and the output, and measuring the total transmission efficiency. Assuming the input and output couplers were fabricated identically, the in- and out-coupling efficiencies to and from the fundamental TE mode in the waveguide should be identical to each other due to the reciprocity theorem. We then measured the coupling efficiency into a multi-mode fiber (MMF) by replacing the fiber in the detection path with a MMF. The laser sweeps are performed using a SolsTiS continuous-wave Ti:Sapphire laser from M Squared. The following equation describes the normalization of our efficiency measurements:

$$\eta_{Coupler} = \sqrt{\frac{P_{PMF/MMF}}{P_{Obj} \cdot \eta_{Setup} \cdot T_{BS} \cdot \eta_{Obj}^2}}, \quad (1)$$

where $\eta_{Coupler}$ is the vertical coupler efficiency, $P_{PMF/MMF}$ is the power in the detection fiber, P_{Obj} is the power in front of the objective, T_{BS} is the transmission at the beamsplitter, and η_{Setup} and η_{Obj} are the losses of setup and objective, respectively. We do not include losses through the

nanobeam that connects two vertical couplers or losses due to coupling into the optical fibers. In our simulations we designed a vertical coupler based on the minimum spot size of our setup, as well as waveguide dimensions given by our cavity design. Thus, increasing the footprint of the vertical couplers did not show improvement in coupling efficiencies once the footprint was larger than the laser spot size. We observed that as soon as the dimensions of the coupler exceed the spot size of the laser, the coupling efficiency does no longer increase. Thus we decided to work with 1.0×1.0 μm^2 vertical couplers. For the current designs we use conservative minimum feature sizes of 100 nm during the device optimization. A decrease in minimum feature size relative to the wavelength would improve the efficiency of the vertical couplers. Furthermore, increasing the laser spot size, device footprint, and waveguide dimensions results in significantly improved efficiencies, which allow us to approach the fundamental limit of $50\ \%$ (as shown in Fig. 5 of the main manuscript).

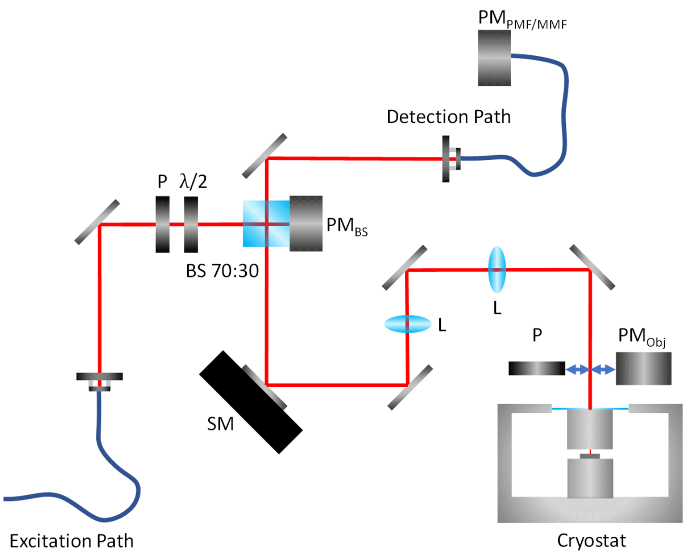

Supplementary Figure 3: **Optical setup.** Optical setup used to characterize the diamond nanophotonic devices. Optical components: polarizer (P), beamsplitter $70 : 30$ (BS 70:30), lenses (L), powermeter (PM), half-wave plate ($\lambda/2$) and scanning mirror (SM).

Supplementary Note 3: Interference at Y-splitter

In Supplementary Figure 4a we tune two cavities into and out of resonance via gas condensation. Three spectra are taken from this measurement and presented in Supplementary Figure 4b. The colors of the data points match the lines shown in Supplementary Figure 4a. We fit the data with Lorentzian line shapes and conclude constructive interference. Using the fit of the red and green curve we calculate the intensity in the case of constructive interference as defined by

$$I_{constr} = I_1 + I_2 + 2\sqrt{I_1 I_2}. \tag{2}$$

Comparing the intensity of the data in blue with the calculated intensities we find:

$$\frac{I_{blue}}{I_{constr,red}} = 1.07 \text{ and } \frac{I_{blue}}{I_{constr,green}} = 0.73, \tag{3}$$

where I_{blue} is the intensity of the data in blue and $I_{constr,red/green}$, the calculated intensity based on the data in red/green. These results suggest a sample drift throughout the experiment. However, when comparing the fit of the blue data with the sum of the detuned peaks we find:

$$\frac{I_{blue}}{I_{sum,red}} = 2.14 \text{ and } \frac{I_{blue}}{I_{sum,green}} = 1.37, \tag{4}$$

where $I_{sum,red/green}$ is the sum of the intensities of the two peaks data in red/green. These results suggest that we see constructive interference and thus that the cavities are approximately in phase and have the same polarization.

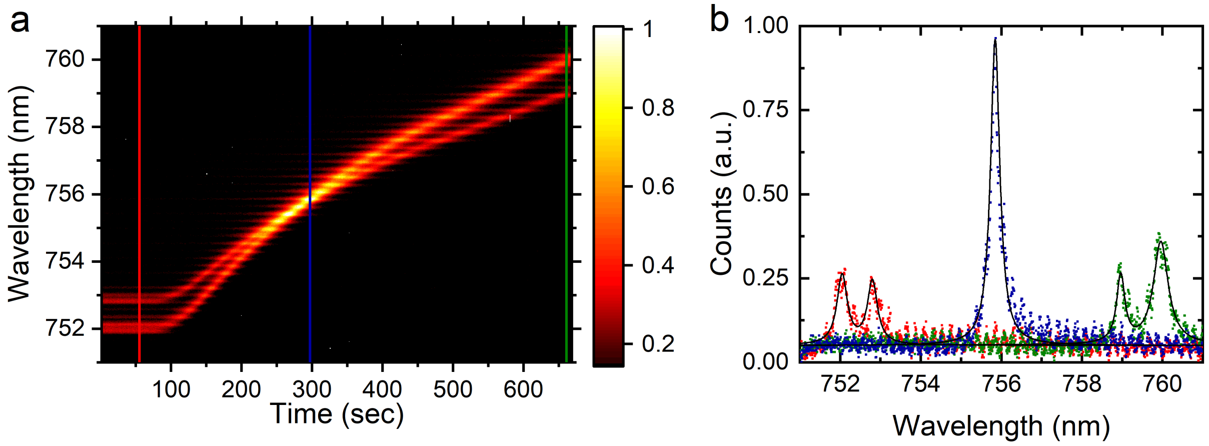

Supplementary Figure 4: **Interference at beamsplitter.** **(a)** Two cavities tune into and out of resonance upon gas condensation. **(b)** Spectra taken from **(a)**, colors correspond to the lines in **(a)**, Lorentzian fit shown in black.

Supplementary Note 4: Gas tuning

In our experiments we use gas tuning, as discussed in the supplementary materials of 1, to shift the resonance of our cavities.[2] During this process we inject about 0.01 sccm of argon gas into our cryostat with typical pressures below 10^{-8} Torr. The argon gas then condenses to our structures at temperatures of 5 K and changes the refractive index at the diamond-air interface. This change in refractive index leads to a red-shift of the resonance frequency of our structures. That the two cavities in Fig. 4 of the main text shift at different speeds is a result of the injection method. We feed a copper tube into our Montana Instruments cryostat and control the flow with a gas mass flow controller. The injection through the tube results in a directionality in the deposition and the fast tuning photonic crystal nanobeam cavity acts as an umbrella for the second structure. By injecting gas in a more uniform way one could tune the cavities more uniformly and could adjust for fabrication related detuning via heating with a non-resonant laser.

Supplementary Note 5: Emitter properties

Through different forms of ion implantation[1,3] and subsequent annealing we can generate silicon-vacancy centers (SiV) prior to or after fabrication. The implantation is performed by CuttingEdge Ions and we follow the annealing procedure described in reference 1, i.e. 4 hours at 400 C, 8 hours at 800 C and 2 hours at 1200 C. A typical photoluminescence spectrum of several SiV centers in a waveguide at 5 K is shown in Supplementary Figure 5a. The emitters show minimal inhomogeneous broadening and a photoluminescence excitation measurement of the C transition of one of the emitters yields a linewidth of $\approx$ 250 MHz (Fig. 5b), which suggests that our fabrication protocols allow for excellent emitter properties.

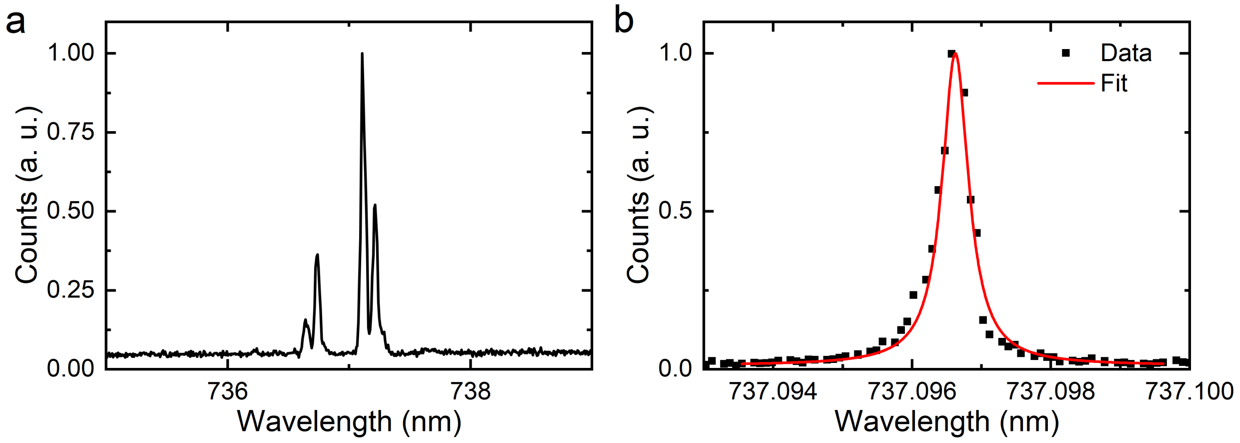

Supplementary Figure 5: **Emitter properties. (a)** Photoluminescence spectrum of several SiVs at 5K using a 720 nm laser for excitation. **(b)** Photoluminescence excitation measurement of the SiV C transition yields a $\approx$ 250 MHz linewidth.

Supplementary References

1. Sipahigil, A. *et al.* An integrated diamond nanophotonics platform for quantum-optical networks. *Science* **354**, 847–850 ((2016)).

2. Strauf, S. *et al.* Frequency control of photonic crystal membrane resonators by monolayer deposition. *Appl. Phys. Lett.* **88**, 043116 ((2006)).

3. Schröder, T. *et al.* Scalable focused ion beam creation of nearly lifetime-limited single quantum emitters in diamond nanostructures. *Nat. Commun.* **8**, 15376 ((2017)).
